# Translation and Cross-Cultural Adaptation of a Validated Questionnaire to Assess Dietary Fiber Intake Among the Italian Population

**DOI:** 10.3390/nu17061084

**Published:** 2025-03-20

**Authors:** Silvia Marconi, Giulia Gilberti, Elisa Romele, Roxanne Barbara Doerr, Anna Simonetto, Monica Marullo, Alice Vetturi, Giacomo Montani, Maurizio Castellano, Barbara Zanini

**Affiliations:** 1 Department of Clinical and Experimental Sciences, University of Brescia, 25123 Brescia, Italybarbara.zanini@unibs.it (B.Z.); 2 Department of Economics and Management, University of Brescia, 25123 Brescia, Italy; 3 Department of Civil, Environment, Architectural Engineering, and Mathematics, University of Brescia, 25123 Brescia, Italy; 4 Department of Molecular and Translational Medicine, University of Brescia, 25123 Brescia, Italy; 5 Dietetics and Clinical Nutrition Service, ASST Spedali Civili of Brescia, 25123 Brescia, Italy

**Keywords:** dietary fiber, validated questionnaire, Italian population, translation, cultural adaptation, health benefits

## Abstract

**Background/Objectives**: Regular consumption of dietary fiber (DF) has been associated with non-communicable disease prevention and health benefits. As a result, having the ability to quickly and correctly estimate DF intake would allow the identification of at-risk groups and the development of public programs aimed at improving their intake. In this study, the authors translate and cross-culturally adapt a validated short food frequency questionnaire for the assessment of DF intake, thus adapting it to the Italian population. **Methods**: The process followed a six-step standardized protocol, and the pre-final version was tested among 40 volunteers. Six experts in nutrition performed the content validity study: Scale-level Content Validity Indexes based on Average (S-CVI/Ave) and Universal Agreement (S-CVI/UA) were calculated (cut-off = 0.83). **Results**: Both S-CVI/Ave and S-CVI/UA were 1.00 ± 0.0, higher than the cut-off value. The preliminary qualitative analysis showed an average DF intake of 21.5 ± 7.2 g/day, which is below the suggested national and international dietary targets, and mainly derives from fruits, vegetables, bread, and cereals. Based on their DF intake, subjects were classified as low (40%), moderate (37.5%), and high (22.5%) consumers. **Conclusions**: The translation and cross-cultural adaptation of a validated short food frequency questionnaire to assess DF intake provided us with a tool suitable for the Italian population. Its application in a real case has enabled the identification of three consumption classes, which can support the design of future studies and interventions.

## 1. Introduction

Suboptimal diets are responsible for millions of deaths and disability in the world, and leading dietary risk factors include insufficient intake of whole grains, nuts, legumes, fruits, and vegetables (as assessed in the Global Burden of Disease Study [1]). All these foods are great providers of dietary fiber (DF), as they are predominantly plant-based carbohydrates that escape the digestion of the small intestine and pass into the large intestine. According to the Codex Alimentarius definition, “*dietary fibres are carbohydrates polymers and oligomers composed by ten or more monomeric units, belonging to the following categories: edible carbohydrate polymers that naturally occurred in the food as consumed; carbohydrates polymers, which have been obtained from food raw material by physical, enzymatic or chemical means; synthetic carbohydrates polymers. All these elements have been shown to have a physiologic effect of benefit to health as demonstrated by generally accepted scientific evidence to competent authorities*” [2]. Methods for separating and quantifying total DF and soluble and insoluble DF have been developed since the early 20th century [3].

Soluble and insoluble DF result in multiple health effects: gut motility stimulation and constipation prevention and treatment, ability to slow down the absorption of carbohydrates and lipids, thus exerting a positive effect on blood glucose and cholesterol levels, improvement and prolongation of the satiating effect of food, and prebiotic action on the intestinal microbiota [4,5]. In a landmark literature review, it was emphasized that a higher intake of total DF was significantly associated with reduced incidence and mortality from several non-communicable diseases (NCD). Cohort studies reported a reduced risk of heart disease incidence and mortality, as well as of diabetes incidence, while prospective studies have shown a reduction in all-cause mortality, total cancer deaths, total cardiovascular disease deaths and several cancer incidences, with dose–response relationships [6].

Recently, the influence of DF in modulating depressive and anxiety outcomes through the microbiota–gut–brain axes has been underlined. Fermentable fibers, particularly those with prebiotic properties as fructans and galacto-oligosaccharides, increase the abundance of beneficial taxa, and in combination with short chain fatty acids (SCFAs) produced by bacteria through the breakdown of fermentable fiber, exert anti-inflammatory activity [7].

The US Food and Drug Administration (FDA) recommends 28 g DF intake per day for an average 2000 kcal/day diet, while the American Dietetic Association recommends a DF amount of 14 g/1000 kcal, or 25 g for adult women and 38 g for adult men [8,9]. According to Italian reference values [10] and the European Commission, the adult population should consume at least 25 g DF per day, even if the energy intake is under 2000 kcal/day. Recommendations differ for children and for the elderly population according to age [10]. Finally, the UK Food Standard Agency recommends 18 g DF intake per day, which is less than most other recommendations [11,12]. Surveys conducted in recent years to assess DF consumption across Europe estimated an average DF consumption of 18–24 g per day among men and 16–20 g per day among women, overall, below most reference intakes [11,12]. Focusing on DF consumption among the Italian population, the actual average intake was estimated to be 20 g per day for males and 17 g per day for females, values below the recommended levels [13].

Considering that modern western diets, which are poor in DF, are rapidly spreading throughout European countries and North America, the inadequacy in DF intake is likely to continue to increase in the future. This reduction in DF is mainly due to the rising consumption of refined cereals, ultra-processed, and ready-to-eat foods, which have a lower fiber content compared to meals prepared from raw ingredients [4,14]. To address this decrease in DF consumption among people following a western diet, a multidimensional approach, including improvement of industrial food processing, the increasing availability of DF rich foods, and even ameliorating dietary and lifestyle education, is required. Nutritional studies aimed at assessing food intake are difficult to conduct for a variety of reasons which include the inaccuracy of self-reporting and the presence of several confounding factors such as differences in diet, cultural habits, and lifestyle. The availability of validated and reliable food questionnaires to correctly estimate different food and nutrient intakes is still one of the hardest challenges in nutritional epidemiology. In the most updated scientific literature, there are few questionnaires dedicated to surveying DF consumption, and they are mainly devoted to the assessment of consumption frequencies of DF-rich foods. Moreover, to accurately assess DF intake and design targeted interventions, the questionnaires must be tailored to the target population [15].

The habitual DF intake short food frequency questionnaire (DFI–FFQ) is a validated and user-friendly questionnaire developed by Professor Genelle Healey and collaborators. The DFI–FFQ is suitable for self-compilation, and it can be applied in cross-sectional, longitudinal, and intervention studies [16]. The questionnaire was designed to quickly quantify grams of DF daily consumption and subsequently classify subjects as low, moderate, and highly habitual DF consumers. This instrument was developed in English-speaking countries so, to assess fiber intake among the Italian population, translation and cross-cultural adaptation were mandatory. A standardized method is needed to achieve the equivalence between the original validated source and the target version of the questionnaire and maintain the content validity [15,17].

The aim of this work was to translate Healey’s DFI–FFQ from English to Italian while culturally adapting and validating the questionnaire to obtain a scientifically solid instrument for assessing DF intake that could be suitable for self-completion among the Italian population [16].

## 2. Materials and Methods

### 2.1. Translation and Cross-Cultural Adaptation Process

**Figure 1:**
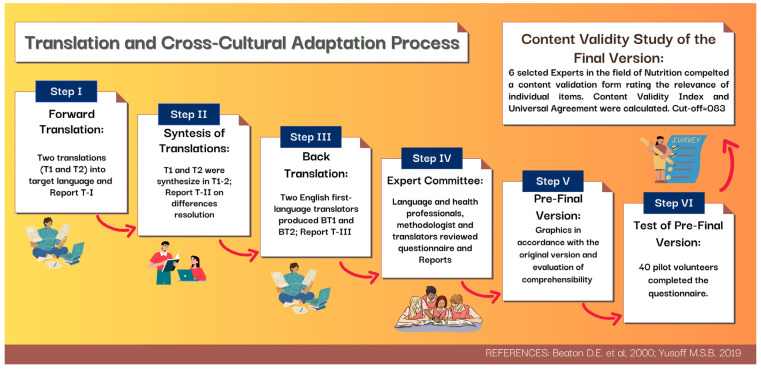
Translation and cross-cultural adaptation process. Description of the different steps that characterize the translation and cross-cultural adaptation process, according to the protocols described by Beaton D.E. and Yousuff M.S.B. [17,18].

Before beginning the translation and cross-cultural adaptation, the authors contacted Professor Genelle Healey, the head of the research team responsible for developing the DFI–FFQ, who granted permission for its use in the present study. Figure 1 outlines the six steps involved the translation and cross-cultural adaptation process, according to a validated algorithm [17,18].

The original version of the questionnaire consists of a short introduction reporting the number of questions and the estimated time of completion. Full name and date of birth are then required; we decided to make these data optional depending on the specific study being conducted, thus respecting the anonymous nature of future studies. Questions are structured in five separate boxes aimed at assessing the consumption frequency of fruit, vegetables, bread and cereals, nuts and seeds, and legumes, respectively; responses range from “never” to “6 or more times per day”. This structure was faithfully respected throughout the process of translation and cross-cultural adaptation.

Step I. Forward Translation: The initial step in the process of adaptation, which involved translating the questionnaire from the source language (English) to Italian, was the “Forward Translation”. This was carried out in February 2024 by two independent bilingual translators, both native speakers of the target language (Translator 1-S.M.: aware of the concepts examined in the questionnaire; Translator 2-S.C.: neither aware nor informed of the concepts examined in the questionnaire). The translators produced two independent translations (T1 and T2) and two written reports of the translation, with additional comments highlighting challenging phrases or uncertainties (Reports T-I; 10 February 2024).

Step II. Synthesis of the Translations: Translators 1 and 2 and a recording Observer (B.Z.) synthesized the results of the two translations (T1 and T2), producing one common Translation (T1-2). The Observer produced a written report documenting the synthesis process and how any divergence was resolved (Report T-II; 13 February 2024).

Step III. Back Translation: Working from the T1-2 version of the questionnaire, two Back Translators, both native speakers of the source language (English) and totally blind to the original version (Back Translator 1-G.O.; Back Translator 2-L.F.), produced two independent back translations (BT1 and BT2). To avoid informational bias, the two Back Translators had no medical background and were not informed about the concepts explored.

The recording Observer (B.Z.) and Translator-1 (S.M.), both with a medical and dietetic background and aware of the concepts being examined in the questionnaire, compared BT1 and BT2 and produced a written report documenting the critical issues underlined by the Back Translators and the differences in the versions produced (Report T-III; 17 April 2024).

Step IV. Expert Committee: To achieve cross-cultural equivalence, an expert Committee was created consisting of a language professional (R.B.D.), a statistician (A.S.), a health professional (B.Z.), and the translators (S.M., S.C., G.O., L.F.). Experts reviewed the original questionnaire, each translation (T1, T2, BT1, BT2), and the corresponding reports (Reports T-I, T-II, T-III). They reached a consensus on any discrepancy and developed the Pre-Final Version. Each decision made by the Committee was documented along with the corresponding reasons. Four areas of equivalence between the original and Pre-Final version were achieved: semantic, idiomatic, experiential, and conceptual (Final Report; 18 April 2024).

Step V. Pre-Final Version: In accordance with the original version of the questionnaire, the division into five sections was maintained, and detailed examples were given of what one serving is equivalent to for each food group according to Italian habitual foods. The frequency of consumption for the average number of servings consumed over the past year was provided as follows: Never, Less than 1 serving per MONTH, 1–3 servings per MONTH, 1 serving per WEEK, 2–4 servings per WEEK, 5–6 servings per WEEK, 1 serving per DAY, 2 servings per DAY, 3 servings per DAY, 4 servings per DAY, 5 servings per DAY, 6 or more servings per DAY (in Italian: Mai, Meno di 1 porzione al MESE, 1–3 porzioni al MESE, 1 porzione a SETTIMANA, 2–4 porzioni a SETTIMANA, 5–6 porzioni a SETTIMANA, 1 porzione al GIORNO, 2 porzioni al GIORNO, 3 porzioni al GIORNO, 4 porzioni al GIORNO, 5 porzioni al GIORNO, 6 o più porzioni al GIORNO).

To ensure the level of comprehensibility, the Pre-Final version was submitted to two 12-year-old volunteers (E.O. and M.C.), who shared their doubts and questions with part of the Expert Committee (B.Z. and S.M.).

Step VI. Test of the Pre-Final Version: The Pre-Final Version underwent the final stage of the translation and cross-cultural adaptation process through its compilation by 40 anonymous pilot volunteers enrolled among the friends and family members of the contributors to this project. Paper questionnaire completion was performed in June 2024, with an average completion time of 10 min. Volunteers were encouraged to provide written comments or suggestions to highlight critical aspects and improve the level of understanding. Single responses, missing items, and suggestions were analyzed by the Expert Committee and used to develop the Final Version (Supplementary Material File S1).

### 2.2. DFI–FFQ Scoring Sheet

In order to calculate the fiber content of traditional Italian foods, modifications were made to the scoring sheet kindly provided by Prof. Healey. Fiber content was calculated using the Table of Food Composition—CREA [19] and the Excel scoring sheet, which was similar to the original and developed to quantify the amount of DF consumed by each participant (Supplementary Material File S1). According to the cut-off proposed by Prof. Healey, individuals were classified as low (females < 18 g/day; males < 22 g/day), moderate (females 18–24.9 g/day; males 22–29.9 g/day) and high (females ≥ 25 g/day; males ≥ 30 g/day) DF consumers [16].

### 2.3. Content Validity Study of the Pre-Final Version

A Content Validity Study (CVS), as previously described, is defined as the degree to which the elements of an assessment questionnaire are relevant and representative for the purpose of the instrument [18]. A CVS was performed on the Final Version of the DFI–FFQ and, given the nature of the questionnaire, the selection of CVS Experts focused on dietitians and nutritionists who were experienced in epidemiological studies. Since the number of Experts defines the Content Validity Index (CVI) cut-off and acceptability, six Experts in the field of nutrition: G.C.V.V., E.R., G.G., M.M., E.C., and A.F.; the acceptable cut-off score for the CVI should be at least 0.83 [18].

Following the CVS protocol, a content validation form was developed and consisted of an introduction for Experts about what is required and a rating scale about the degree of relevance (1 = the item is not relevant to the measured domain; 2 = the item is somewhat relevant to the measured domain; 3 = the item is quite relevant to the measured domain; 4 = the item is highly relevant to the measured domain). Experts were asked to assign a score from 1 to 4 for each item of the questionnaire; subsequently, the relevance rating was recorded as 1 for scores 3 or 4, and as 0 for scores 1 or 2 [15]. A general evaluation on the clarity of the instructions, the time required for completion, and the overall adequacy of the questionnaire for the measures it was designed to assess was then requested. Subsequently, the CVS Experts were asked to provide written comments to improve the relevance of the items.

### 2.4. Data Analysis

The validation of the survey content was verified for each item of the questionnaire (I-CVI), indicating the percentage of agreement among the Experts, with a preset cut-off of S-CVI/Ave and S-CVI/UA = 0.83 deemed acceptable [18].

For preliminary data about DF intake, according to the Pre-Final version of the questionnaire among volunteers, the analysis included descriptive statistics (i.e., frequencies and percentages for categorical variables and mean values with standard deviations for continuous variables) and was performed using STATA (Stata Statistical Software: Release 16.0 College Station, TX, USA: Stata Corporation); graphs processing was performed with Microsoft Excel (2016).

### 2.5. Ethical Considerations

The Pre-Final Version of the questionnaire was anonymously completed by volunteers who were authorized to withdraw their participation from the survey at any stage. To ensure the confidentiality of volunteers, an anonymous paper-format questionnaire was used, and all procedures of the study complied with the provisions of the General Data Protection Regulation (GDPR 679/2016). Due to the anonymous nature of this survey, personal and sensitive data could not be traced by the researchers and, consequently, the study protocol did not require any ethics committee approval.

## 3. Results

The process of the translation and cross-cultural adaptation involved the compilation of the instructions for the questionnaire, food items, serving size, and frequencies of consumption. Each step of the process, documented in the different Reports (drafted in Italian and available on request) led to the resolution of all divergences in the translation process and generated the Pre-Final Version of the Italian DFI–FFQ. Between May and June 2024, the CVS Experts submitted their responses. The relevance rating assigned by each individual Expert for each item was recorded in an Excel file and then used to calculate the following indices:

- Experts in Agreement = sum of the relevant rating provided by all Experts for each item.
- I-CVI = item-level content validity index = (Experts in Agreement)/(number of Experts).
- S-CVI/Ave = scale-level content validity index based on the average = Average of I-CVI; CVI can also be calculated as the average of proportion relevance score for each individual Expert.
- Universal Agreement (UA) = 100% Expert in agreement provided a relevance rating for each item. Dichotomous variable indicating perfect agreement among all experts for each item = 1, if all Experts provided a relevance rating of 1, or 0, if at least one Expert provided a relevance rating of 0 for the considered item.
- S-CVI/UA = scale-level content validity index based on universal agreement = Average of UA.

The scores obtained from the CVI study (Table 1), and the suggestions shared by the 6 nutrition Experts were used for changes to the Final version.

The global assessment of the questionnaire demonstrated that the Content Validity Index reached values higher than cut-off 0.83: specifically, both S-CVI/Ave and S-CVI/UA averages and standard deviations were 1.00 ± 0.00 (Table 1). In addition, the I-CVI of all individual items was always 1.00, above the cut-off level, and the opinions provided by nutrition Experts in the open-ended questions confirmed the clarity of all contents, as well as their quick compilation and suitability for the present purpose.

Step VI of the process required the Pre-Final version of the questionnaire to be completed by a pilot group of volunteers who were native Italian speakers; in this project, 40 subjects, consisting of 20 females and 20 males, were enrolled. The mean age of the population was 46.4 ± 19.2 years (range 20–84 years) and four different generations were included: Boomers—before 1964, 10 subjects (25%); X Generation—born from 1965 to 1980, 12 subjects (30%); Millennials—born from 1981 to 1996, 8 subjects (20%); Z Generation—born from 1997 to 2012, 10 subjects (25%) [20].

**Figure 2:**
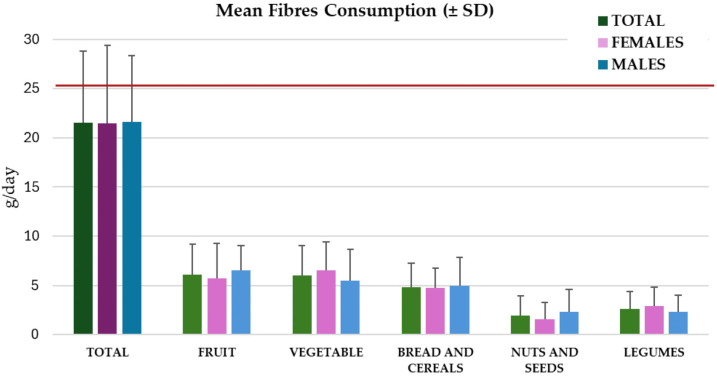
Mean dietary fiber intake for the total population, differentiating between sources and gender.

Preliminary data collected from the qualitative evaluation of these questionnaires showed the following: The mean dietary fiber intake for the total population was 21.5 ± 7.2 g/day, mainly from fruits (6.1 ± 3.1 g/day), vegetables (6.0 ± 3.0 g/day), and bread and cereals (4.9 ± 2.4 g/day). Nuts and seeds and legumes provided 1.9 ± 2.0 g/day and 2.6 ± 1.8 g/day, respectively. A comparison of the answers given by the female and male populations showed a *p*-value > 0.05. The mean DF consumption was 21.5 ± 7.9 g/day among females and 21.6 ± 6.7 g/day among males; there was a tendency among females to consume more DF from vegetables and legumes than the male population, which consumes more DF from fruits, nuts, and seeds (Figure 2).

Categorizing the total population according to the cut-off values applied by Prof. Healey, 40% of our pilot group are low (30% females and 50% males), 37.5% are moderate (40% females and 35% males), and 22.5% are high DF consumers (30% females and 15% males).

**Figure 3:**
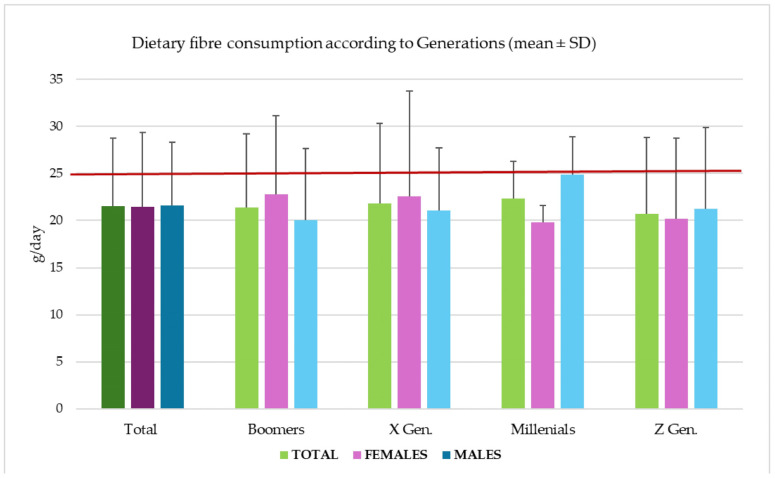
Mean dietary fiber consumption according to Generations (mean ± SD). X Generation—born from 1965 to 1980, 12 subjects (30%); Millennials—born from 1981 to 1996, 8 subjects (20%); Z Generation—born from 1997 to 2012, 10 subjects (25%) [20].

In analyzing the sample according to the Generations breakdown, a slight variation in DF consumption was observed among groups, with a trend toward reduced consumption in the Z Generation. Differential analysis between females and males indicated a higher DF consumption among males only in the Millennials generation, whereas there was a trend toward higher DF consumption among females in earlier generations (Figure 3).

Even when comparing the DF consumption of the pilot group and differentiating by gender or generation, DF intakes were lower than those suggested by the Italian Reference values of LARN (at least 25 g/day; red line Figure 2 and Figure 3; [10]).

## 4. Discussion

To our knowledge, there are currently no validated short questionnaires specifically developed for quantifying DF intake in the Italian population. In most of the currently available studies, food diaries or extensive food frequency questionnaires are used to estimate DF intake. A validated, easy-to-use, and quick tool that can assess the actual DF intake could be a valuable instrument for nutrition experts for both clinical and research settings.

The main objective of this study was to translate, cross-culturally adapt, and validate a short food frequency questionnaire about DF intake, starting from a previously validated English version [16], through an assessment by experts and a subsequent implementation of a pilot test among Italian adults. To ensure the congruence between the study objective and the data collection instrument, the content validity index (CVI) was used and researchers found excellent validity of the items both individually (I-ICVI) and overall (S-CVI-Ave); moreover, the experts expressed universal agreement [17,18]. The performance of a test on the Pre-Final version was an essential step to ensuring that the adapted version retained its equivalence in an applied situation. Additionally, quantifying DF intake in a small representative sample of the Italian population provides a basis for evaluating its methodological and procedural aspects in view of future larger-scale studies.

Non-communicable diseases account for 71% of global deaths, and poor-quality diets characterized by high levels of salt, sugar, fats, and a low intake of fiber, represent the greatest risk factors [21,22]. Insufficient DF intake is one of the preventable dietary risk factors leading to chronic diseases such as Type 2 diabetes, cardiovascular disease, and colorectal cancer [1,6]. In contrast, positive effects of DF on disease modulation include fermentability, the prebiotic effect, absorption capacity, hydration, and viscosity property, which also result in increased satiety, better glycemic and lipid control, and improved bowel health [23]. This body of evidence allows the conclusion that increasing DF intake should improve population health [6,24].

Despite the scientific evidence of the last decades supporting the multiple health benefits of DF, and the recommendation of food-based dietary guidelines worldwide, current dietary habits in Europe and the western world still lack in fiber. The European Food Safety Authority recommends a DF intake of 25 g/day for adults and Italian guidelines suggest an intake of 12.6–16.7 g/1000 kcal, with a daily target of 25 g [10]. According to research data, amounts greater than 30 g per day confer additional health benefits [6]. A 2017 study shows that only Germany and Hungary are close to reaching the recommended DF intake levels, while all other countries across Europe have lower consumption. Italy, with an estimated DF intake of 19.6 g/day for males and 17.7 g/day for females, is positioned at the end of the European rankings [11]. Data from a more recent survey by CREA, updated in January 2024, showed an overall DF amount of 17 g/day of fiber among the general Italian population. Focusing on adults between 18 and 64 years old, a DF intake of 20 g/day and of 17 g/day for males and for females was recorded, respectively. Among the elderly population (>65 years old), slightly higher intakes were reported (24 g/day and 19 g/day, respectively), but they remain below recommended values [13,19,25].

In our small pilot sample, the data are partially in line with the national and international trends, with higher consumption in males than in females only in the youngest segment of the population (subjects born before 1981—Millenials). Among Generation X and Boomers (people born before 1980), females seem to consume more fiber than males. Future studies, conducted in large population groups, will allow these preliminary observations to be verified. Overall, the lowest fiber consumption was observed in younger individuals born after 1997. As in other studies among Italian adults, the main sources of DF are fruits, vegetables, bread, and cereals, while legumes, nuts, and seeds contribute only a small amount [26]. In this small sample, females have higher DF consumption overall compared to the national average (21.5 ± 7.9 g/day vs. 17 g/day), as do males (21.6 ± 6.7 g/day vs. 20 g/day), but given the small sample size, only preliminary evaluations are possible.

Strength and Limitations: The present study provides the first simple, quick, and validated instrument to quantify DF intake suitable among the Italian population that has proven its very satisfactory internal reliability and validity. A heterogeneous team of experts supervised the translation, the trans-cultural adaptation procedure, and the content validity index analysis, making this work scientifically valuable. All the procedures were conducted in line with a validated algorithm, and, in accordance with the protocol, a team with different areas of competence was involved. The major limitation of the present study lies in the small number of volunteers that tested the Pre-Final version, which nevertheless made it possible to conduct a row descriptive analysis. In addition, in conformity with the rules on data protection and privacy, no socio-economic, anthropometric measures, or other sensitive data were collected. Although articles on the translation and adaptation of this questionnaire have already been published in the literature [15], this new tool will hopefully be useful in clinical settings and for future research studies assessing eating habits and different lifestyle aspects among the Italian population.

## 5. Conclusions

The promotion of dietary fiber consumption through messages that help improve food selection by detailing its benefits for the prevention of chronic non-communicable diseases and a healthier quality of life is a major global issue. Wide-ranging projects require appropriate tools adapted to the target population and characterized by linguistic and cultural appropriateness. This translated and cross-culturally adapted validated short food frequency questionnaire for the Italian population may represent a first step in improving future interventions.

## Figures and Tables

**Table 1:** Content Validity Index Study and Relevance Ratings on the item scale by Experts (*n* = 6).

The Relevance Ratings of the Item Scale by Experts										
	1—GCVV	2—ER	3—GG	4—MM	5—EC	6—AF		Experts in Agreement	I-CVI	UA
Item										
1	1	1	1	1	1	1		6	1	1
2	1	1	1	1	1	1		6	1	1
3	1	1	1	1	1	1		6	1	1
4	1	1	1	1	1	1		6	1	1
5	1	1	1	1	1	1		6	1	1
Proportion relevance	1	1	1	1	1	1		S-CVI/Ave	1	
								S-CVI/UA		1
Average proportion of item judged as relevance across the 6 Experts							1			

## Data Availability

Each step of the process of translation and cross-cultural adaptation, documented in the different Reports and drafted in Italian, will be available on request.

## Supplementary Materials

The following supporting information can be downloaded at: https://www.mdpi.com/article/10.3390/nu17061084/s1, Supplementary Material File S1. a. DFI-FFQ Original English-Version; b. DFI-FFQ Final-Version, drafted in Italian; Table S1: Comparison of fiber intakes.

## Abbreviations

The following abbreviations are used in this manuscript:

DF	Dietary fiber
DFI-FFQ	Dietary fiber intake–short food frequency questionnaire
CVI	Content Validity Index
I-CVI	Item-level content validity index
S-CVI	Scale-level content validity index
UA	Universal agreement
