# Supplementary material for: Translation and Cross-Cultural Adaptation of a Validated Questionnaire to Assess Dietary Fiber Intake Among the Italian Population
Source: Nutrients. 2025 Mar 20;17(6):1084. doi: 10.3390/nu17061084 (PMC11944576; doi:10.3390/nu17061084)
Supplement: Supplementary file 1 [file nutrients-17-01084-s001.zip › nutrients-3511457-Supplementary Material.pdf]

Supplementary Material File S1. a. DFI-FFQ Original English-Version; **b**. DFI-FFQ Final-Version, drafted in Italian.

a.

Supplementary Materials: Validity and Reproducibility of a Habitual Dietary Fibre Intake Short Food Frequency Questionnaire

Genelle Healey, Louise Brough, Rinki Murphy, Duncan Hedderley, Chrissie Butts and Jane Coad

Introduction

* Welcome to the Dietary Fibre Intake Short Food Frequency Questionnaire (DFI-FFQ).

The questionnaire contains 5 questions about your FOOD INTAKE and is estimated to take you around 5-10 MINUTES to complete.

Please enter your FULL NAME in the box below.

* Please also enter the DATE and MONTH of your birthday in the BOX BELOW, e.g. 2nd July

Fruit intake

On average, over the PAST YEAR, how many serves of FRUIT have you consumed?

The following are examples of 1 SERVE of FRUIT

1 medium piece of fruit- i.e. apple, banana, orange, tomato or pear OR
2 small pieces of fruit- i.e. apricot, kiwifruit or plum OR
1/2 cup fresh, frozen, tinned or stewed fruit- i.e. berries or tinned peaches OR
1 small handful of dried fruit- i.e. sultanas

	Never	Less than 1 serve per MONTH	1-3 serves per MONTH	1 serve per WEEK	2-4 serves per WEEK	5-6 serves per WEEK	1 serve per DAY	2 serves per DAY	3 serves per DAY	4 serves per DAY	5 serves per DAY	6 or more serves per DAY
Fruit	◯	◯	◯	◯	◯	◯	◯	◯	◯	◯	◯	◯

Vegetable intake

On average, over the PAST YEAR, how many serves of VEGETABLES have you consumed?

The following are examples of 1 SERVE of VEGETABLES

1 medium potato, kumara, yam, taro or carrot OR
1/2 cup cooked broccoli, green peas, corn, pumpkin or spinach OR
1 cup salad

	Never	Less than 1 serve per MONTH	1-3 serves per MONTH	1 serve per WEEK	2-4 serves per WEEK	5-6 serves per WEEK	1 serve per DAY	2 serves per DAY	3 serves per DAY	4 serves per DAY	5 serves per DAY	6 or more serves per DAY
Vegetables												

Bread and Cereal intake

On average, over the PAST YEAR, how many serves of BREADS AND CEREALS have you consumed?

The following are examples of 1 SERVE of BREADS AND CEREALS

Wholegrain/wholemeal- 1 slice of bread, 1 small roll or 1 wrap OR
White- 2 slices of bread, 2 small rolls or 2 wraps OR
Rice/pasta- 1/2 cup cooked brown rice or wholemeal pasta or 1 cup cooked white rice or white pasta OR
Cereals- 1/2 cup cooked porridge or muesli, 1/3 cup All/Sultana/San Bran or 2 Weetbix

	Never	Less than 1 serve per MONTH	1-3 serves per MONTH	1 serve per WEEK	2-4 serves per WEEK	5-6 serves per WEEK	1 serve per DAY	2 serves per DAY	3 serves per DAY	4 serves per DAY	5 serves per DAY	6 or more serves per DAY
Breads and Cereals												

Nut and Seed intake

On average, over the PAST YEAR, how many serves of NUTS AND SEEDS have you consumed?

The following are examples of 1 SERVE of NUTS AND SEEDS

2 tablespoons of peanut butter OR
1/3 cup (or a small handful) of nuts or seeds (e.g. cashew nuts, almonds, pistachio nuts, brazil nuts, macadamia nuts, hazel nuts, chia seeds, sunflower seeds, pumpkin seeds, sesame seeds)

	Never	Less than 1 serve per MONTH	1-3 serves per MONTH	1 serve per WEEK	2-4 serves per WEEK	5-6 serves per WEEK	1 serve per DAY	2 serves per DAY	3 serves per DAY	4 serves per DAY	5 serves per DAY	6 or more serves per DAY
Nuts and Seeds	○	○	○	○	○	○	○	○	○	○	○	○

Legume intake (e.g. beans, peas and lentils)

On average, over the PAST YEAR, how many serves of LEGUMES (e.g. BEANS, PEAS and LENTILS) have you consumed?

The following are examples of 1 SERVE of LEGUMES (e.g. BEANS, PEAS and LENTILS)

3/4 cup tofu OR
3/4 cup cooked legumes (e.g. kidney beans, chickpeas, green/brown/red lentils, hummus, baked beans, split peas, canned bean mix, broad beans, white/black beans)

	Never	Less than 1 serve per MONTH	1-3 serves per MONTH	1 serve per WEEK	2-4 serves per WEEK	5-6 serves per WEEK	1 serve per DAY	2 serves per DAY	3 serves per DAY	4 serves per DAY	5 serves per DAY	6 or more serves per DAY
Legumes-Beans, peas and lentils	○	○	○	○	○	○	○	○	○	○	○	○

Figure S1. Example of the habitual dietary fibre intake short food frequency questionnaire which was generated using SurveyMonkey.

b.

QUESTIONARIO BREVE DELLE FREQUENZE DI CONSUMO DI FIBRE ALIMENTARI

Introduzione:

Benvenuta,

Benvenuto,

il “Questionario Breve delle Frequenze di Consumo di Fibre Alimentari” è composto da 5 domande riguardanti le Sue abitudini di consumo di alcuni alimenti e la compilazione richiederà all’incirca 5-10 minuti.

Per cortesia, inserisca il Suo anno di nascita e selezioni il genere nei riquadri sottostanti:

□ Femmina
□ Maschio
□ preferisco non dichiarare

Consumo di Frutta

In media, durante l’ultimo anno, quante porzioni di FRUTTA ha consumato?

Di seguito alcuni esempi su cosa si intende per “1 PORZIONE DI FRUTTA”:

1 frutto di medie dimensione (per esempio mela, banana, arancia o pera) OPPURE

2 frutti di piccole dimensioni (per esempio albicocca, kiwi o prugna) OPPURE

½ coppetta di frutta fresca, surgelata, in scatola o cotta (per esempio frutti di bosco o pesche in scatola) OPPURE

1 manciata di frutta disidratata (per esempio uva sultanina)

	Mai	Meno di 1 porzione al MESE	1-3 porzioni al MESE	1 porzione a SETTIMANA	2-4 porzioni a SETTIMANA	5-6 porzioni a SETTIMANA	1 porzione al GIORNO	2 porzioni al GIORNO	3 porzioni al GIORNO	4 porzioni al GIORNO	5 porzioni al GIORNO	6 o più porzioni al GIORNO
Frutta												

Consumo di Verdura

In media, durante l’ultimo anno, quante porzioni di VERDURA ha consumato?

Di seguito alcuni esempi di cosa si intende per “1 PORZIONE DI VERDURA”:

1 patata di medie-grandi dimensioni, 2-3 carote o 2-3 zucchine OPPURE

½ piatto di broccoli cotti, fagiolini o spinaci cotti OPPURE

1 ciotola grande di insalata

	Mai	Meno di 1 porzione al MESE	1-3 porzioni al MESE	1 porzione a SETTIMANA	2-4 porzioni a SETTIMANA	5-6 porzioni a SETTIMANA	1 porzione al GIORNO	2 porzioni al GIORNO	3 porzioni al GIORNO	4 porzioni al GIORNO	5 porzioni al GIORNO	6 o più porzioni al GIORNO
Verdura												

Consumo di Pane e Cereali

In media, durante l'ultimo anno, quante porzioni di PANE E CEREALI ha consumato?

Di seguito alcuni esempi di cosa si intende per "1 PORZIONE DI PANE E CEREALI":

Pane:

Se integrale: 1 fetta di pane, 1 panino piccolo o 1 piadina piccola OPPURE

Se non integrale: 2 fette di pane, 1 pane grande o 1 piadina grande OPPURE

Riso/pasta:

Se integrale: 2-3 cucchiai di riso o pasta a cotto OPPURE

Se non integrale: 5-6 cucchiai di riso o pasta a cotto OPPURE

Cereali:

Se integrali: 3-4 cucchiai di cereali di avena cotti (tipo porridge), cereali con crusca o muesli, cereali soffiati OPPURE

Se non integrali: 5-6 cucchiai di cereali di avena cotti (tipo porridge), cereali o muesli, cereali soffiati

	Mai	Meno di 1 porzione al MESE	1-3 porzioni al MESE	1 porzione a SETTIMANA	2-4 porzioni a SETTIMANA	5-6 porzioni a SETTIMANA	1 porzione al GIORNO	2 porzioni al GIORNO	3 porzioni al GIORNO	4 porzioni al GIORNO	5 porzioni al GIORNO	6 o più porzioni al GIORNO
Pane e Cereali												

Consumo di Frutta Secca a Guscio e Semi

In media, durante l'ultimo anno, quante porzioni di FRUTTA SECCA A GUSCIO E SEMI ha consumato?

Di seguito alcuni esempi di cosa si intende per "1 PORZIONE DI FRUTTA SECCA A GUSCIO E SEMI":

1 manciata di frutta secca a guscio o semi (per esempio anacardi, mandorle, noci, pistacchi, noci del Brasile, noci macadamia, nocciole, semi di chia, semi di zucca, semi di sesamo) OPPURE

3 cucchiai di crema a base di frutta secca a guscio (per esempio burro di arachidi)

	Mai	Meno di 1 porzione al MESE	1-3 porzioni al MESE	1 porzione a SETTIMANA	2-4 porzioni a SETTIMANA	5-6 porzioni a SETTIMANA	1 porzione al GIORNO	2 porzioni al GIORNO	3 porzioni al GIORNO	4 porzioni al GIORNO	5 porzioni al GIORNO	6 o più porzioni al GIORNO
Frutta Secca a Guscio e Semi												

Consumo di Legumi (es. fagioli, piselli e lenticchie)

In media, durante l'ultimo anno, quante porzioni di LEGUMI (es. fagioli, piselli e lenticchie) ha consumato?

Di seguito alcuni esempi di cosa si intende per "1 PORZIONE DI LEGUMI (es FAGIOLI, PISELLI e LENTICCHIE):

> 4-5 cucchiai di legumi cotti (per esempio, fagioli, ceci, lenticchie verdi/marroni/rosse, hummus, piselli, mix di legumi in scatola, fave, lupini, cicerchie, soia, fagioli azuki) OPPURE
>
> 2 fette di derivati della soia come tofu o tempeth

	Mai	Meno di 1 porzione al MESE	1-3 porzioni al MESE	1 porzione a SETTIMANA	2-4 porzioni a SETTIMANA	5-6 porzioni a SETTIMANA	1 porzione al GIORNO	2 porzioni al GIORNO	3 porzioni al GIORNO	4 porzioni al GIORNO	5 porzioni al GIORNO	6 o più porzioni al GIORNO
Legumi												

La ringraziamo per la Sua preziosa collaborazione.

Se durante la compilazione alcuni quesiti risultassero poco chiari, o avesse osservazioni da condividere, ce lo può segnalare nelle righe sottostanti. Un sentito grazie.

____

____

____

Table S1. Comparison of fibre intake Healey's questionnaire and Italian references (Healey G 2016; Italian Society of Human Nutrition- SINU 2014).

Comparison of fibre intake; Healey questionnaire vs Italian version

Food	Fibre content (g/100g)	Servings (according to LARN)	Fiber content (g/serving)	Servings according to Healey G.	Average fibre intake for 1 serving (g)
apple (peel-free)	1.7	150g	2.6	1 medium piece	
banana	1.8	"	2.7	"	3.06
orange	1.6	"	2.4	"	
pear without peel	3.8	"	5.7	"	
apricot	1.56	"	2.35	2 small pieces	
kiwi	2.2	"	3.3	"	
plum	1.5	"	2.25	"	
blueberries	3.1	"	4.65		
raisin	5.2	30g	1.56	1 small handful	
potatoes	1.6	200g	3.2	1 mediumpotato/taro/yam	
carrots	3.1	200g; 2-3	6.2	"	3.89
zucchini	1.3	"	2.6	"	
broccoli	3.1	"	6.2	1/2 cup cooked	
green beans	2.9	"	5.8	"	
pumpkin	0.5	"	1	"	
spinach	2.3	half plate	4.6	"	
lattuce	1.9	large bowl; 80g	1.52	1 cup	
white bread (00 type)	3.2	100g	3.2	2 slices	
wholemeal bread	6.5	50g	3.25	1 slice	
cooked pasta	0.9	200g	1.8	1 cup cooked	
cooked whole wheat pasta (half portion)	3.5	90g	3.15	1/2 cup cooked	2.21
cooked rice	0.2	260g	0.5		
cooked brown rice (half portion)	0.8	125g	1		
muesli	6.4	30g	1.92		

mixed cereal flakes	6.5	"	1.95		
porridge	8.8	"	2.64	1/2 cup of cooked	
whole grain granola	8.6	"	2.58	1/3 cup	
whole grain cereals	7.6	"	2.28	1/3 cup	
cashews	3	30g	0.9	1/3 cup or small hadful	
almonds	12.7	"	3.81		
walnuts	6.2	"	1.86		3.46
hazelnuts	8.1	"	2.43		
pistachios	10.6	"	3.18		
macadamia nuts	8.6	"	2.58		
Brazil nuts	7.5	"	2.25		
chia seeds	37.7	"	11.31		
sesame seeds	14	"	4.2		
peanut butter	6.8	30g	2.04		
cooked legumes (canned)	6.8	150g/half plate	10.2	3/4 cup of cooked	
canned chickpeas	8.1	"	12.5		
canned lentils	5.3	"	7.95		7.61
dried chickpeas	13.8	50g/3-4 spoons	6.9		
dried beans	17	"	8.5		
dried lentils	13.7	"	6.85		
tempeth	2.8	2 slices	5.6		
tofu	1.2	2 slices	2.4	3/4 cup	
